# Satisfaction with vaccination services and its relationship to emotional responses of service users in Lima. LEGADO’s quality management model as a public solution to promote citizen emotional well-being during pandemic

**DOI:** 10.3389/fpubh.2023.1136312

**Published:** 2023-08-07

**Authors:** Agustin Espinosa, Jordi Marti, Alicia Calderón-Prada, Milagros Ticliahuanca, Jacqueline Lobrano, Nataly Carreón

**Affiliations:** ^1^Departamento de Psicología, Grupo de Psicología Política y Social de la Pontificia Universidad Católica del Perú, Pontificia Universidad Católica del Perú, Lima, Peru; ^2^Proyecto Especial Legado de los Juegos Panamericanos y Parapanamericanos Lima, Lima, Peru

**Keywords:** emotional responses, public services, SARS-CoV-2, satisfaction with vaccination, emotional well-being

## Abstract

This article analyzes the levels of citizen satisfaction with LEGADO’s quality management model service during the first year of vaccination against SARS-CoV-2 in public spaces administered by LEGADO, and its relationship with the user’s emotional responses. To this end, a survey study has been developed from July 2021 until March 2022 at 4 moments to citizens (*n* = 1,697) who attended 3 vaccination locations administered by LEGADO (VIDENA, Complejo VMT and Polideportivo VES). The results show a high level of satisfaction with LEGADO’s quality model service, which is associated with a positive emotional balance. Specifically, the elements that have the greatest effect on positive emotions are the cleanliness and facilities’ organization and the agility of service. These results are discussed emphasizing the importance of the role of public institutions in developing inclusive quality public services for all citizens. This strategy of public quality model service according to citizens’ necessities should result in confidence towards public institutions and socially responsible behavior among citizens through the reduction of social gaps. The research establishes the urgency to promote this model in order to bring legitimacy and confidence to public institutions in Perú.

## Health system collapse in Peru during pandemic

Peru has been one of the countries most affected by the pandemic produced by the accelerated global spread of the SARS-CoV-2 virus (1–3). From the first case officially detected in the country on March 6, 2020 (4) to November 27, 2021, 660,000 infections were officially diagnosed, with 200,000 deaths as a consequence of the disease (5). In absolute terms, the mortality rate reached in the country, placed it in December 2021 as the sixth country with the highest number of deaths, behind the United States, Brazil, India, Mexico and Russia (6). However, a contextualized analysis introducing the mortality rate per million inhabitants, Peru ranked as the most affected country with 616.2 deaths per million inhabitants by June 2021 (7).

The country’s precarious public health system was a major impediment to successfully addressing a complex health situation like the pandemic (2). As an example, prior to the health crisis, Peru had only 946 Intensive Care Unit (ICU) beds, resulting in a total of 29 beds per million inhabitants. This demonstrates the scarcity of these materials in a country whose health-care system is based on hospitals rather than primary and preventative care (2). Additionally, a disarticulated health system (8) reveals problems in coordination across different levels of government.

In the described scenario, and in order to limit the spread of the virus and avoid the.

collapse of the health system, Martin Vizcarra, the former President of the Republic, declared a nationwide state of emergency on March 11, 2020 (9), which became effective on March 16 and was characterized by a quick and firm response to the potential risks posed by the virus (10). This state of emergency initially included regulations such as mandatory social immobilization and quarantines for citizens, along with behavioral measures aimed at preventing contagion, such as mandatory use of masks in public spaces, practice of social distancing, and use of disinfectant alcohol, among others (11).

However, due to issues related to Peruvian social structure, such as informality, household overcrowding, poor provision of water and sanitation services, and the precariousness of health-care delivery (12), the measures implemented failed to prevent the health-care system from collapsing (1). This resulted in the country’s high rates of infection and mortality (1), primarily among citizens with lesser economic wealth, underlining social inequities. Furthermore, the pandemic’s economic and social crisis exacerbated the condition mentioned above, resulting in a decrease in employment and labor income, as well as an increase in the rate of precarious and informal employment (13). As a result, complaints and doubts arose, both within Peruvian society and in international spheres, about the country’s functioning and institutional efficacy, as well as its capacity to manage the health emergency appropriately (1).

As a result of the aforementioned asymmetries, citizens have been affected in different degrees by the pandemic; citizens from lower socioeconomic strata, lower educational levels, more exposed to employment informal economy, and with less access to health services, have suffered a greater number of infections and deaths attributed to SARS-CoV-2 (14).

Concerning the situation described, the Political Constitution of Peru reaffirms the right of all citizens to the protection of their health (15), and more broadly, the National Centre of Strategic Planning (16) in its country’s vision towards 2050, sets as objectives the achievement of reaching “peoples’ potential in equal opportunities without discrimination to enjoy a fulfilling life” (p.2) and the construction of a “modern, efficient, transparent and decentralized State that guarantees a fair and inclusive society without corruption and leaving no one behind” (pp.2–3). Although the pandemic has shown that these objectives are still far from being accomplished. According to the Instituto Nacional de Estadística e Informática (17) while the Peruvian State has shown an average annual growth of 4,8% of its gross domestic product between 2000 and 2019, there is still much to improve in areas such as the provision of public services and the regulation of certain private activities. Thus, institutional precariousness and poor public administration produce a vicious circle in which crisis situations are aggravated. As a consequence, there is an urgent need to develop a set of political reforms aimed at defending the public sector in order to build a State that guarantees the exercise of rights for all Peruvians (18, 19).

## LEGADO and the vaccination process: a public commitment to strengthening citizen emotional well-being

The citizenry and other political and social actors responded to institutional weakness and the widespread crisis caused by the epidemic in a variety of ways that sometimes aggravated the negative impacts of the crisis situation. In the context mentioned above, it is not unexpected that distrust of the state and its institutions is growing. As a result of institutional delegitimization, society experiences a negative socio-emotional climate of demoralization and helplessness toward the system, which leads to citizen disinterest in public affairs (see (20)). Indeed, the State’s structural deficiencies cause major inequities in access to services, undermining the social contract, that is, the disconnection between the State and the citizens, who do not find genuine support to develop and improve their emotional well-being in the former. This circumstance exacerbates the sense of being orphaned in the community, as citizens believe that improving their quality of life is solely dependent on their own efforts (12).

Given this scenario, the task is to strive for a long-term vision of the country, one that is committed to an agenda that defends the public interest, which requires substantive reform of the political system and institutions (18). This reform must be consistent with a public value approach that can generate trust and legitimacy towards institutions, promote citizen participation, guarantee equity, strengthen the social fabric, and ensure social and environmental sustainability, as processes that contribute to the common good, guaranteeing principles and procedures of justice and equity (21). Nevertheless, distrust of the State and its institutions affects the reform processes. Distrust is seen in statistics such as Latinobarómetro (22), which shows that only 13% of the Peruvian population trust the government. In fact, when discussions on how vaccination process should be implemented arise in Perú in 2021, some political actors and members of civil society advocate for a private intervention, based in a perceived better performance of this sector to the detriment of public entities (23, 24).

As previously stated, this viewpoint is built on a perception of disconnection and distrust toward the state and public institutions; as a result, the private sector gains greater value at expense of the public sector. This rhetoric was reiterated by some presidential candidates in 2021 who advocated for vaccine acquisition under private management (23, 24). This position, however, contradicts international evidence showing that, in the face of complex health crises, nations have the strongest logistical capability and ability to respond to population requirements (see (25)). In this regard, before weakening the state’s role, it is critical to ensure the strengthening of public health, expanding service supply, and modernizing management (12), as a means of prevention and care in the face of health emergencies such as the SARS-CoV-2 pandemic, and in order to generate emotional well-being, equity, and social trust, among other social benefits (26).

For the purpose of building an agenda to safeguard the public interest over the private one, it should be recognized that, despite the conflict of interests between the private and the public, the role of the State in the vaccination process was reaffirmed through the acquisition and provision of several vaccines against SARS-CoV-2 as a health public strategy (27). One of the governmental actors committed to the vaccination process has been the Proyecto Especial Legado Juegos Panamericanos y Parapanamericanos Lima 2019 (from here on LEGADO), which, by governmental mandate, institutionally commits its organizational structure and its capacities for the operation and logistical monitoring and support in various actions aimed at mitigating the negative effects of the pandemic in Peru (28).

It should be noted that LEGADO was originally created to ensure the maintenance and proper use of infrastructure, as well as the provision of quality services through the capacity gained in the organization and public administration of the Pan American and Parapan American Games Lima 2019, thereby creating social value and emotional well-being for Lima citizens (29). However, in the context of the pandemic, LEGADO added the following to its duties in an extraordinary way by government order: (1) management of care and temporary isolation centers, (2) acquisition and implementation of oxygen plants, and (3) logistical support to the SARS-CoV-2 vaccination campaign in some Lima vaccination facilities (30). As a result, LEGADO has played an important role in serving the public since May 2021, initially with three vaccination centers under its supervision: (1) the Villa Deportiva Nacional (from here on VIDENA), (2) the Polideportivo Villa El Salvador (from here on Polideportivo VES), and (3) the Complejo Deportivo Andrés Avelino Cáceres de Villa María del Triunfo (from here on Complejo VMT) (30), subsequently providing logistical support to a total of 32 vaccination centers in the city of Lima (28).

## Public administration, quality of service and citizen emotional well-being

In a scenario difficult to build an acceptable degree of trust in institutions, it is a priority to develop policies and strategies to promote institutional legitimacy. This is necessary in the elaboration of a national health policy consistent with the constitutional mandate and the country’s development objectives (15, 16). This is important to enable citizen commitment for the public’s good defense. In this sense, studies such as that of Palacios et al. (31) shows that levels of institutional trust in Peru were directly associated with citizen willingness to comply with the norms proposed by the government during the pandemic. On the contrary, the reasons for non-compliance are diverse and range from a set of irrational beliefs to people’s necessity to subsist in the face of the economic crisis caused by the health emergency that affected Peruvian society. The fact that the monetary transfer program was consistently delayed due to a lack of adequate records of potential beneficiaries and citizens’ low levels of financial inclusion – less than half of the population had a bank account - meant that people continued to work informally and that most beneficiaries collected their subsidies and monetary aids in person, which contributed to keeping the contagion curve on the rise (32). In any case, there is a deficient institutional structure to guarantee basic emotional well-being conditions for important sectors of the population (31).

Gilles et al. (33), on the other hand, discovered in a study in Switzerland that trust in public health institutions is directly associated with the success of SARS-CoV-2 vaccination process. To put it another way, the more citizens trust health organizations, the more likely they are to follow official vaccination and protection measures (33). Based on this evidence, institutional reform is required to place the citizen at the core of the State’s priorities in order to build a country with opportunities for all (12), as well as to allow the State to establish a national dialog with the private sector from a firm and consolidated position, supported by solid institutions (34).

Aligned with this, and with the goal of reducing the pandemic’s consequences in the short term, it was important to develop an efficient and effective vaccination program focused on citizens’ needs, perceived satisfaction and emotional well-being. Serving this purpose, LEGADO developed a quality management model service strategy for the vaccination process based on lessons gathered during the recent Pan American and Parapan American Games Lima 2019.

Practices that differentiate LEGADO apart from other public institutions in Peru are based in a special normative framework given to special public projects that were established by the Ministry of Economy and Finance (Ministerio de Economía y Finanzas in spanish). This framework has been exemplified by the efficiency of New Engineering-type contracts (NEC), which are contracts designed to provide clarity and flexibility in the execution of public projects (35).

In practice, these characteristics resulted in the following actions: (1) to develop and train members of an organization in pro-sociality and productivity resources; (2) to work using management tools that facilitate the members of the organization to understand the interrelationship that exists between their tasks with other areas of the organization, as well as to organize the development of procedures and processes, (3) to promote a healthy work environment, based on a systemic optimism that gives people the energy, conviction and empowerment to perform their tasks responsibly and does not lead to burnout or optimism bias, and (4) promoting a leadership style with pro-social and productive characteristics, in such a way that the project leaders are drivers of change and inspiration for their teams and the rest of the organization, proposing, based on innovation, the implementation of some specific management tools required by the organization (36).

In this way, citizens’ demands and needs were placed at the center of institutional decisions, with the scope of monitoring them by satisfaction and emotional well-being assessments for continual service improvement. As such, LEGADO’s service strategy implies an improvement of physical spaces and care processes that could produce what Ramkissoon (37) calls place affect, which is defined as “the emotional bonding an individual forms with a place” (p.5) which in turn should promote emotional well-being, greater trust in institutions and a greater predisposition of citizens to vaccination processes (38).

Regarding vaccination, it is well known that, at the beginning of pandemic, misleading propaganda in social media through fake news, pretends to install suspicion around vaccines. It resulted in an increase of Peruvians who did not want to be vaccinated from 20% in August 2020 to 40% in December 2020 according to National polls (39). Taking in consideration this continuous distrust around COVID vaccines, LEGADO’s quality model service was conceived for the citizens’ satisfaction and emotional well-being in order to increase vaccination rates among the population. Regarding satisfaction with health services, Burt et al. (40) found in Australia that citizens satisfied with vaccination services administered by health professionals, showed greater willingness to receive a vaccine in the future under similar conditions. On the other hand, Barrera et al. (41) found that the national immunization program in Guatemala had achieved remarkable progress in this area of public health, since 70.4% of users of vaccination campaigns reported the service as “good” or “very good,” while only 4% as “bad” or “very bad,” thus showing high levels of citizen satisfaction. Taking into account the aforementioned, satisfaction measurement is a crucial factor that should be studied and incorporated to guarantee the proper functioning of the service provided, as well as to improve the service by correcting the deficiencies identified (42), and thus facilitate the rapprochement between the State and the citizens.

Considering all of the above, it is important to highlight that the relationship between citizens and public institutions in Peru is based on distrust. In this sense, as proposed by Ramkissoon (43), interventions on public services that promote positive relationships between citizens and public institutions, such as those services developed by LEGADO, could help improve trust in institutions, as well as promote the emotional well-being of citizens.

In this scenario, it is also important to investigate the level of emotional well-being of service users. One of the approaches to well-being is that of subjective well-being, which can be defined as an individual’s continuous perception that his or her life is good and pleasant (44). The study of subjective well-being comprises three components: positive affect, negative affect and life satisfaction (45, 46). The first two are affective in nature and the third involves a cognitive judgment about the living conditions to which a person is exposed (47). It has been consistently found that those who report greater positive affectivity report greater satisfaction with their life and specific aspects of it (48). It is the emotional component of subjective well-being that is evaluated in the present study.

It’s also relevant to mention that, in order to accomplish these measuring objectives, LEGADO’s research team convened with the academic sector. In this case, LEGADO achieved an alliance with Pontifical Catholic University of Perú, specifically with the Political and Social Psychology Research Team (Grupo de Psicología Política y Social, GPPS by its initials in spanish) belonging to the Psychology Department. This alliance between the public and academic sector is unusual and innovative by itself, because it is focused in bringing theory framework and methodology validity to research in public topics, and for the academic sector it allows access to citizens’ samples to enrich theoretical analysis. Which was the case for this particular study, introducing the methodology assessment to safeguard the validity and reliability of the research and also, psychological variables for emotional well-being indicators.

Based on the above, the goal of this study is to describe and analyze the levels of satisfaction according to LEGADO’s quality model service developed for the vaccination process, and its relationship with emotional responses as an indicator of users’ emotional well-being, at four different moments of the vaccination stages. These are as follows: at the end of July 2021 (when vaccination was aimed at citizens over 40 years old), in mid-September 2021 (when vaccination was aimed at citizens over 25 years old, and additional booster doses were given to other groups), between November and December 2021, and finally between March and April 2022 (when vaccination was already being given to adolescents and booster doses continued to be given to older groups).

Considering the above, the hypothesis is that there will be a positive relationship between satisfaction with the vaccination service and emotional well-being. In other words, emotional well-being, measured by positive and negative emotional responses, will be enhanced by satisfaction with the service.

## Methods

### Participants

The participants of the present study were 1,697 citizens of Lima Metropolitana distributed by sex in 877 women (51.7%) and 820 men (48.3%), whose ages ranged from 0 to 90 years old (*M* = 37.52, SD = 17.15). This sample was taken from 4 different moments of the vaccination process, during the years 2021 and 2022. Specifically, 407 participants (24%) were selected from July 13 to July 21, 2021, 433 participants (25.5%) from September 21 to September 28, 2021, 434 participants (25.5%) from November 29 to December 6, 2021 and; finally, 423 participants (25%) from March 28 to April 4, 2022. In the case of people under 18 years of age and adults with difficulty answering (due to any kind of handicap), the accompanying person was considered to answer the survey. Therefore, two questionnaires were designed, one for the accompanying person and the other for the person directly vaccinated. When people under 18 years of age were selected, the accompanying individual was approached directly; in the case of adults with any handicap that made it difficult to answer, they were asked if they wished to participate directly or if the accompanying person could be asked.

In total, most of the surveys were carried out at the VIDENA, with 625 respondents (36.8%), followed by the Polideportivo VES with 540 participants (31.8%), and finally the Complejo VMT with 532 cases (31.4%). Table 1 shows the detailed distribution of participants by moment and place of vaccination.

**Table 1:** Participants of the study by moment and place of vaccination.

		By moment of vaccination				
		First moment	Second moment	Third moment	Fourth moment	Total
By vaccination center	VIDENA	183	159	170	113	625
	Complejo VMT	120	134	129	149	532
	Polideportivo VES	104	140	135	161	540
	**Total**	**407**	**433**	**434**	**423**	**1,697**

### Procedure

First, the dates for the questionnaire’s four application moments were determined. At each level of data collection, cluster sampling was performed, with each vaccination center representing a cluster. The sample size was defined at each time point with a 95% confidence level and an approximate margin of error of 5%, taking the total population of the four sites as an infinite population (or of size larger than 10,000 cases) (49). As a result, representative samples of approximately 400 cases were obtained for each stage of the data collection, by means of simple random sampling, which were then distributed proportionally by cluster (see Table 1).

An interviewer addressed the participants who were located at the randomly selected spots for the application of the questionnaires at the vaccination locations. Regarding ethical aspects, participants were presented with an informed consent protocol and were informed that their participation in the study was anonymous, voluntary and that the data would be analyzed collectively. Likewise, when questionnaires were applied to under-age participants, an assent protocol was applied to them, in addition to requesting the consent of their parents or tutors so that they could complete the questionnaire. It should be noted that these questionnaires were applied by LEGADO volunteers previously trained in the methodology and handling of the survey.

### Measurement

#### Sociodemographic data

The questionnaire used included a sociodemographic form that collected pertinent data about the participants, such as their sex, age, and district of residence, among other variables. Furthermore, information on the vaccination process was acquired, such as the presence of a companion, the kind of entry to the vaccination place (pedestrian or vehicle access), and the vaccination place (VIDENA, Complejo VMT, Polideportivo VES).

#### Satisfaction with the vaccination service

With respect to the satisfaction of the participants with the vaccination service, 11 items were designed based on a Customer Service Journey approach, which evaluates the customer’s experience throughout the entire process (50). All of these items reflect different aspects of this service administered by LEGADO to serve the public. The evaluated aspects include the following points: (1) Citizen security around the vaccination center, (2) waiting time to enter the vaccination center, (3) order in the lines for people/cars outside the vaccination center, (4) application of biosecurity controls upon entry, (5) ease of movement within the vaccination center (provision of ramps, wheelchair loans, wide spaces, etc.), (6) cleanliness inside the vaccination center, (7) waiting time to be vaccinated inside the vaccination center, (8) order in the distribution, location of spaces and comfort (chairs, table, sunshades, order of parking lots, lavatories, toilets), (9) signs to identify the different areas and/or spaces (entrance, exit, toilets, topic, vaccination areas, etc.), (10) courtesy of the personnel inside the vaccination center and (11) clarity of information within the vaccination center. These items consist of a score from 1 to 5, where 1 is “totally dissatisfied” and 5 is “totally satisfied.” In addition to the above, a question on the general satisfaction with the vaccination process, with the same response scale, was also added.

#### Emotional well-being – emotional responses towards vaccination service

The concept of subjective well-being has been widely used as equivalent to life satisfaction and positive/negative emotionality, since these concepts refer to a basic emotional feeling about the evaluation of the quality of personal life (45, 51). Furthermore, subjective well-being can distinguish in generic terms, the cognitive aspects of well-being, represented by the assessment of life satisfaction, and the affective or emotional aspects, represented by the affective balance or personal experience of positive/negative emotions (45, 52, 53). As previously mentioned, it is the emotional component of subjective well-being that is measured in the present study.

A list of emotions was selected and evaluated in the present study after a review of several emotion scales (54–56). As a result, a list of 7 emotions was obtained, 3 positive and 4 negative, which were relevant to inquire about the affective state of the citizens surveyed in relation to the vaccination process in the face of the pandemic.

These emotions were: (1) joy, (2) hope, (3) sadness, (4) anger, (5) fear, (6) anxiety and (7) pride, in which it was asked how much each of these emotions was felt with respect to their experience with the vaccination process. For each emotion, responses were recorded on a scale from 1 to 5, where 1 means “not at all” and 5 means “very much.” The objective of this specific scale was to measure the emotions of the participants at that precise moment in time (directly after vaccination). For the purposes of the analysis, each of these emotions is reported separately, although additionally an indicator of positive emotions was constructed, formed by the arithmetic mean of the scores on joy, pride and hope, and an indicator of negative emotions based on the arithmetic mean of the scores on anger, fear, anxiety and sadness. Subsequently, an indicator of the balance of emotions was made, which is the subtraction of the score of the positive emotions minus the negative emotions. This balance of emotions can be interpreted by cataloging the value obtained from the formula described above, where a negative or zero value indicates a bad emotional balance and positive values show a good one. An emotional scale was used because in psychological literature, it is agreed that emotions are an acceptable way to assess well-being (53).

## Results

In general, results on satisfaction show a good evaluation of LEGADO’s performance during the vaccination process. Table 2 displays the percentages of satisfaction with several aspects of the vaccination service by vaccination moment and location. The evaluated aspects that result in a better overall performance (percentage of people satisfied/very satisfied with the service) include: courtesy of the personnel inside the vaccination center (96.9%), clarity of information within the vaccination center (96.4%), order in the distribution, location of spaces and comfort (chairs, table, sunshades, order of parking lots, lavatories, toilets) (95.7%), ease of movement within the vaccination center (provision of ramps, wheelchair loans, wide spaces, etc.) (94.6%) and, cleanliness inside the vaccination center (94.3%). It should be noted that the additional aspects evaluated also showed high percentages of satisfaction, being in all cases higher than 79%. However, there were fluctuations in some of these scores by moment and place of vaccination (see Table 2).

**Table 2:** Levels of satisfaction (% satisfied or totally satisfied) with different aspects of the service provided by LEGADO during the vaccination process.

	By moment of vaccination				By vaccination center			
	First moment	Second moment	Tercer moment	Fourth moment	VIDENA	Complejo VMT	Polideportivo VES	Total
Citizen security around the vaccination center	90.9%	91.4%	88.7%	92.2%	88.7%	93.0%	91.1%	90.8%
Waiting time to enter the vaccination center	92.9%	79.4%	86.6%	94.4%	87.9%	91.3%	85.6%	88.2%
Order in the lines for people/cars outside the vaccination center	94.8%	84.5%	90.3%	93.7%	90.9%	92.5%	89.0%	90.7%
Application of biosecurity controls upon entry	80.6%	87.0%	82.0%	97.6%	79.7%	91.6%	91.9%	86.9%
Ease of movement within the vaccination center	95.3%	95.6%	94.5%	93.0%	93.1%	95.1%	95.8%	94.6%
Cleanliness inside the vaccination center	96.5%	97.0%	93.3%	90.3%	90.4%	97.8%	95.4%	94.3%
Waiting time to be vaccinated inside the vaccination center	96.6%	84.6%	85.0%	94.8%	90.8%	90.6%	88.9%	90.1%
Order in the distribution, location of spaces and comfort	94.9%	96.8%	94.4%	96.7%	95.3%	96.6%	95.2%	95.7%
Signs to identify the different areas and/or spaces	92.2%	95.0%	92.2%	92.0%	93.1%	92.8%	92.4%	92.8%
Courtesy of the personnel inside the vaccination center	96.8%	97.4%	96.0%	97.4%	97.1%	96.8%	96.9%	96.9%
Clarity of information within the vaccination center	96.8%	97.0%	95.1%	96.7%	96.5%	95.8%	96.8%	96.4%
**General satisfaction with the vaccination process**	**93.5%**	**91.4%**	**90.7%**	**94.4%**	**91.2%**	**94.0%**	**92.6%**	**92.5%**

The means and standard deviations of the emotional responses reported by the participants directly after their vaccination process are also presented, by moment and place of vaccination (see Table 3). Considering the above, in general terms, the participants of the study express emotional well-being right after their vaccination process, showing a positive balance of emotions, although towards the third moment of the data gathering, an increase of negative emotions and a reduction of positive emotions can be appreciated. In spite of this, the values of negative emotions were never higher than those of positive emotions.

**Table 3:** Descriptives (mean and standard deviation) of the emotional responses to the vaccination service provided by LEGADO.

	By moment of vaccination				By vaccination center			
	First moment	Second moment	Third moment	Fourth moment	VIDENA	Complejo VMT	Polideportivo VES	Total
Joy	*M =* 4.79 S*D* = 0.55	*M* = 4.65 S*D* = 0.68	*M* = 4.45 S*D* = 0.94	*M* = 4.37 S*D* = 0.95	*M* = 4.64 S*D* = 0.78	*M* = 4.72 S*D* = 0.68	*M* = 4.32 S*D* = 0.92	*M* = 4.56 S*D* = 0.82
Hope	*M* = 4.69 S*D* = 0.70	*M* = 4.52 S*D* = 0.85	*M* = 4.11 S*D* = 1.27	*M* = 4.03 S*D* = 1.13	*M* = 4.28 S*D* = 1.18	*M* = 4.59 S*D* = 0.84	*M* = 4.15 S*D* = 1.03	*M* = 4.34 S*D* = 1.05
Sadness	*M* = 1.60 S*D* = 1.10	*M* = 1.49 S*D* = 1.00	*M* = 1.82 S*D* = 1.24	*M* = 1.93 S*D* = 1.25	*M* = 1.86 S*D* = 1.33	*M* = 1.59 S*D* = 1.09	*M* = 1.65 S*D* = 1.01	*M* = 1.71 S*D* = 1.17
Anger	*M* = 1.38 S*D* = 0.91	*M =* 1.42 S*D* = 0.92	*M* = 1.70 S*D* = 1.11	*M* = 1.94 S*D* = 1.27	*M* = 1.71 S*D* = 1.23	*M* = 1.49 S*D* = 0.99	*M* = 1.61 S*D* = 0.98	*M* = 1.61 S*D* = 1.09
Fear	*M* = 1.71 S*D* = 1.20	*M* = 1.58 S*D* = 1.04	*M* = 1.91 S*D* = 1.29	*M* = 1.98 S*D* = 1.32	*M* = 1.89 S*D* = 1.32	*M* = 1.72 S*D* = 1.23	*M* = 1.76 S*D* = 1.10	*M* = 1.80 S*D* = 1.24
Anxiety	*M* = 1.75 S*D* = 1.26	*M* = 1.64 S*D* = 1.16	*M* = 1.98 S*D* = 1.34	*M* = 1.99 S*D* = 1.30	*M* = 1.87 S*D* = 1.30	*M* = 1.88 S*D* = 1.38	*M* = 1.77 S*D* = 1.12	*M* = 1.84 S*D* = 1.27
Pride	*M* = 4.63 S*D* = 0.81	*M* = 4.32 S*D* = 0.1.09	*M* = 3.78 S*D* = 1.49	*M* = 3.59 S*D* = 1.43	*M* = 3.92 S*D* = 1.48	*M* = 4.48 S*D* = 1.06	*M* = 3.84 S*D* = 1.22	*M* = 4.07 S*D* = 1.30
Positive emotions	*M =* 4.71 S*D* = 0.52	*M* = 4.50 S*D* = 0.67	*M* = 4.11 S*D* = 1.01	*M* = 4.00 S*D* = 0.89	*M* = 4.28 S*D* = 0.92	*M* = 4.60 S*D* = 0.66	*M* = 4.10 S*D* = 0.85	*M* = 4.32 S*D* = 0.85
Negative emotions	*M* = 1.61 S*D* = 0.78	*M* = 1.53 S*D* = 0.76	*M* = 1.85 S*D* = 0.98	*M* = 1.96 S*D* = 1.11	*M* = 1.83 S*D* = 1.05	*M* = 1.67 S*D* = 0.87	*M* = 1.70 S*D* = 0.86	*M* = 1.74 S*D* = 0.94
**Balance of emotions**	***M* = 3.09 S*D* = 1.03**	***M* = 2.96 S*D* = 1.11**	***M* = 2.26 S*D* = 1.49**	***M* = 2.04 S*D* = 1.50**	***M* = 2.44 S*D* = 1.46**	***M* = 2.92 S*D* = 1.16**	***M* = 2.40 S*D* = 1.41**	***M* = 2.58 S*D* = 1.38**

In a second stage of the analysis, the correlations between the levels of satisfaction and the emotional responses expressed by the participants directly after their vaccination were processed. At the general level, joy was the emotion most closely related to satisfaction with the vaccination service, being observed to correlate positively with the 11 evaluated satisfaction aspects, identifying low to medium-low effect size relationships (0.059 < *r* < 0.172). Hope was positively correlated with 9 satisfaction indicators, with low and medium-low effect sizes (0.051 < *r* < 0.141). Sadness, on the other hand, was inversely associated with 7 indicators of satisfaction, with medium to low effect sizes (−0.213 < *r* < −0.061).

Anger was negatively correlated with 10 satisfaction indicators, with relationships whose effect sizes ranged from medium to low (−0.258 < *r* < 0.053). Fear was negatively associated with 5 indicators, with relationships whose effect sizes ranged from medium-low to low (−0.204 < *r* < −0.059). Anxiety, on the other hand, reported the lowest number of correlations, with only 2 inverse associations, with low effect sizes (−0.149 < *r* < −0.059). Finally, pride reported associations with 8 satisfaction indicators, with low effect sizes (0.051 < r < 0.151) (see Table 4).

**Table 4:** Pearson-type correlations between emotional responses (columns) and satisfaction (rows) in the general sample.

	**Joy**	**Hope**	**Sadness**	**Anger**	**Fear**	**Anxiety**	**Pride**	**Positive emotions**	**Negative emotions**	**Balance of emotions**
Citizen security around the vaccination center	**0.172** **	**0.115** **	−0.044	**−0.064** **	−0.007	−0.020	0.044	**0.126** **	−0.041	**0.105** **
Waiting time to enter the vaccination center	**0.129** **	0.055*	0.001	−0.053*	−0.014	−0.015	0.043	**0.086** **	−0.025	**0.070** **
Order in the lines for people/cars outside the vaccination center	**0.078** **	0.038	**−0.065** **	**−0.074** **	−0.045	0.003	**0.099** **	**0.092** **	−0.055*	**0.094** **
Application of biosecurity controls upon entry	0.059*	−0.002	−0.023	−0.001	0.012	0.019	−0.016	0.010	0.003	0.004
Ease of movement within the vaccination center	**0.070** **	0.051*	**−0.076** **	**−0.098** **	−0.030	−0.005	**0.151** **	**0.122** **	**−0.063** **	**0.118** **
Cleanliness inside the vaccination center	**0.137** **	**0.141** **	**−0.213** **	**−0.258** **	**−0.204** **	**−0.149** **	**0.096** **	**0.152** **	**−0.258** **	**0.268** **
Waiting time to be vaccinated inside the vaccination center	**0.163** **	**0.101** **	−0.004	**−0.081** **	0.013	−0.002	**0.087** **	**0.139** **	−0.021	**0.100** **
Order in the distribution, location of spaces and comfort	**0.126** **	**0.076** **	**−0.090** **	**−0.077** **	−0.059*	−0.030	**0.096** **	**0.122** **	**−0.079** **	**0.129** **
Signs to identify the different areas and/or spaces	**0.103** **	0.053*	−0.061*	**−0.097** **	**−0.065** **	−0.015	0.059*	**0.085** **	**−0.073** **	**0.102** **
Courtesy of the personnel inside the vaccination center	**0.141** **	**0.083** **	**−0.090** **	**−0.154** **	**−0.103** **	−0.059*	0.051*	**0.106** **	**−0.126** **	**0.151** **
Clarity of information within the vaccination center	**0.137** **	**0.093** **	**−0.088** **	**−0.120** **	**−0.071** **	−0.013	**0.072** **	**0.120** **	**−0.090** **	**0.134** **
**General satisfaction with the vaccination process**	**0.213** **	**0.125** **	−0.034	**−0.112** **	−0.041	−0.001	**0.100** **	**0.172** **	−0.056*	**0.144** **

** The correlation is significant at the 0.01 level (bilateral).

*The correlation is significant at the 0.05 level (bilateral).

Specifically, in the first moment, carried out between July 13 and 21, 2021, it was found that the emotion that correlated with the highest number of satisfaction items was joy, presenting a positive association with all indicators except the waiting time to enter the vaccination center. Hope presented a positive correlation with citizen security, application of biosecurity controls and identification signs; while anxiety showed positive correlations with order in the lines, application of biosecurity controls and ease of transfer. In this measurement, none of the satisfaction items were associated with sadness, anger, fear and pride.

In the second moment, carried out from September 21 to 28, 2021, a greater number of correlations can be seen among different emotions and the levels of satisfaction with the vaccination process. In the cases of joy and pride, all satisfaction indicators reported positive correlations with these emotions. On the other hand, hope reported positive correlations with citizen security, entry time, order in the lines, ease of movement, cleanliness at the vaccination place, waiting time, order in the distribution, identification signs, courtesy of the personnel and clarity of information. Sadness was found to be negatively associated with satisfaction with citizen security, cleanliness, order in distribution, identification signs, friendliness of personnel and clarity of information. Anger showed negative correlations with all satisfaction indicators. Fear presented a moderate level of associations, presenting correlations with citizen security, order in the lines, cleanliness, identification signs, courtesy of the personnel and clarity of information. Similarly, anxiety was negatively associated with three indicators of satisfaction, these being citizen security, the application of biosecurity controls and cleanliness at the vaccination place.

The third moment, conducted from November 29 to December 6, 2021, reported the lowest number of associations between emotions and satisfaction indicators. Joy, hope, anger and pride did not present relationships with satisfaction indicators. Fear and anxiety presented positive correlations with waiting time. Sadness was inversely associated with ease of movement within the facilities and positively associated with waiting times for vaccination.

Regarding the fourth and last moment, carried out between March 28 and April 4, 2022, it can be seen that joy showed a positive correlation with 9 items. These were citizen security, waiting time to enter the vaccination center, application of biosecurity controls, cleanliness, waiting time to be vaccinated, order in distribution, identification signs, courtesy of the personnel and clarity of information. Hope showed positive associations with 7 indicators of satisfaction, these being citizen safety, waiting time for entering, application of biosecurity controls, cleanliness, waiting time for being vaccinated, courtesy of the personnel and clarity of information. On the other hand, sadness presented negative correlations with all the items, except with citizen security and ease of movement. Anger also presented the same pattern of negative correlations as sadness, being associated with admission time, order in the lines, application of biosecurity controls, cleanliness, waiting time for being vaccinated, order in the distribution, identification signs, courtesy of the personnel, and clarity of information. Fear reported negative correlations with the items of admission time, application of biosecurity controls, cleanliness, waiting time for being vaccinated, order in distribution, identification signs, courtesy and clarity of information. Anxiety presented negative correlations with admission time, order in the lines, application of biosecurity controls, cleanliness, waiting time for being vaccinated, order in the distribution, identification signs, courtesy and clarity of information. Pride turned out to be the emotion with the least number of correlations with respect to the satisfaction indicators, these being admission time, order in the lines, ease of movement and order in the distribution.

In addition, a stepwise linear regression was performed where all indicators of satisfaction with the vaccination process (except the application of biosecurity controls) were selected as independent variables and positive emotionality was selected as the dependent variable. This analysis was processed in order to observe the joint effect of different aspects of satisfaction with vaccination on positive emotional responses. As a result, 3 models were calculated. Regarding the third and last model obtained, it was significant, *F* (3,1,624) = 20.914, *p* < 0.001, in addition to having a multiple R of 0.19, which explains 3.5% of the variance of positive emotionality. The results of the model obtained can be seen in Table 5.

**Table 5:** Model and regression coefficients predicting the effect of indicators of satisfaction with the vaccination process (IV) on positive emotionality (DV).

Model	Unstandardized coefficients		Standardized coefficients	*T*	Significance	95% confidence interval for B	
	B	error dev.	Beta			Lower limit	Upper limit
(Constant)	2.941	0.176		16.748	0.000	2.597	3.286
Cleanliness inside the vaccination center	0.118	0.030	0.101	3.911	0.000	0.059	0.178
Waiting time to be vaccinated inside the vaccination center	0.096	0.027	0.091	3.537	0.000	0.043	0.149
Citizen security around the vaccination center	0.082	0.028	0.077	2.992	0.003	0.028	0.136

Similarly, a stepwise linear regression analysis was performed where all indicators of satisfaction with the vaccination process (except the application of biosecurity controls) were selected as independent variables and the negative emotionality was selected as the dependent variable. As a result, 2 models were obtained. The second and last model obtained was significant, *F* (2, 1,627) = 62.855, *p* < 0.001, with a multiple R of 0.268, which explains 7.1% of the variance of the negative emotionality. The results can be seen in Table 6.

**Table 6:** Model and regression coefficients predicting the effect of indicators of satisfaction with the vaccination process (IV) on negative emotionality (DV).

**Model**	**Unstandardized coefficients**		**Standardized coefficients**	***T***	**Significance**	**95% confidence interval for B**	
	**B**	**error** **dev.**	**Beta**			**Lower limit**	**Upper limit**
(Constant)	3.129	0.172		18.149	0.000	2.791	3.468
Cleanliness inside the vaccination center	−0.359	0.032	−0.278	−11.180	0.000	−0.422	−0.296
Waiting time to be vaccinated inside the vaccination center	0.068	0.029	0.059	2.351	0.003	0.011	0.125

## Discussion

The objective of this research was to analyze the levels of satisfaction with LEGADO’s quality management model service developed for the vaccination process and its relationship with emotional responses, as indicators of subjective well-being, in the users of this service. Considering this goal, a core element in the present discussion will be to frame the results obtained with the need to strengthen the role of the State in the elaboration of a public health policy from a public value perspective that responds to what is established by the Constitución Política del Perú (15) and by the Centro Nacional de Planeamiento Estratégico (16) in its objectives for the development of the country, which seek to guarantee the right of all citizens to health services that ensure their emotional well-being.

At a general level, the results show that the satisfaction with LEGADO’s quality management model service in vaccination centers, is directly associated with a positive balance of emotions. However, it is important to highlight that this relationship between the dimensions of satisfaction and emotional responses is not consistent over time, considering the different contexts in which the evaluations have been carried out. Thus, on the one hand, at the descriptive level, it can be seen that the levels of satisfaction, although always high, decreased as the vaccination process progressed. A similar trend was observed in the balance of emotions, which, although always positive, began to decrease as the measurements progressed in time.

The reasons for this time variability may be diverse. On the one hand, the greater satisfaction and more positive emotional balance at the first moments of evaluation could be associated with the ease that the vaccination process brought to a society hit hard by the pandemic. It is worth noting that, in early stages of the vaccination process, the people who mostly attended vaccination centers did so voluntarily and according to their age group as scheduled by the government. Regarding them, one of the limitations of the research was the variability of the age of the participants and, if applicable, the lack of control of the age of the companions who answered the questionnaire. Nonetheless, a correlation analysis examined the effect of age variability and found, especially in the first and third measurements, a positive low effect size relationship between age and positive emotionality (0.102 < *r* < 0.160). In the case of the fourth measurement, age was found to be associated with negative emotions with a low effect size (*r* = 0.202).

The aforementioned limitation does not detract from the possibility of interpreting the results of the positive relationship between age and emotional well-being in the first waves of vaccination, as a form of protection for the groups considered at that time to be more vulnerable (the older adult). This result is reversed in the last wave of vaccination evaluated, where the process becomes mandatory for all citizens regardless of their attitudes towards vaccination.

Mainly, study shows that, highest levels of satisfaction and highest prevalence of positive emotions are observed in moments where citizens attended voluntarily and, in their cohort group, when scheduled by government. Subsequent evaluation moments already comprise a habituation of the users with the services that, although they did not lose quality, were already seen as normal by the citizens, and, consequently, this would bring a natural decrease in the satisfaction level curve after the first service evaluations (see (57)).

In addition, in subsequent evaluations, some people may have felt forced to be vaccinated, due to the mandatory nature of the regulation on carrying the vaccination card for access to various services and facilities nationwide. The latter could even have a negative impact on satisfaction and emotional responses to vaccination, as it could be perceived as a violation of the people’s right to decide (58). The decrease in satisfaction and positive emotionality coincided with the third moment of evaluation, when a proportion of people sought vaccination services outside of the State’s scheduled date for them, but within the framework of the mandatory presentation of the vaccination card.

The results also show that satisfaction levels vary significantly depending on the vaccination facility. In fact, Complejo VMT, one of the establishments where low-income citizens attended, has the highest levels of satisfaction and the best balance of emotions, alongside Polideportivo VES. Indicators relating to the quality of the infrastructure and its maintenance are among the aspects of the service best evaluated by individuals who visited the Complejo VMT. Thus, aspects such as the cleanliness of the center, the courtesy of the people who work there, the organization of the processes to receive the service, and the clarity of the information demonstrate the importance of making people feel welcome in spaces that provide warm, quality, and dignified service, regardless of their social status. In contrast, satisfaction ratings are lower at the VIDENA facility, where people from less vulnerable sectors come, though they remain high. These results, the fact that satisfaction with LEGADO’s model service is high regardless of location, demonstrates the success in quality public administration practice with inclusive policy. In other words, all citizens receive high-quality services without prejudice to their origin or social status. On the other hand, the fact that satisfaction levels are slightly higher in those who attend the facility located in a suburban region traditionally poorer, can be interpreted as a positive assessment of a health service, with an unusual quality for citizens of these population areas (59, 60). A key element of this result is that LEGADO, with its model management service during the vaccination process in Lima, has achieved the goal of generating quality spaces and services for those who previously had limited access to them (29).

The satisfaction expressed by users of the vaccination service includes an evaluation of specific aspects that, as a whole, show how the good functioning of a public service positively influences relations among citizens. In fact, it is not a minor issue that, among the most valued aspects are the courtesy of the workers, the clarity of the information in the establishment and the organization during the vaccination process. Hence, the positive evaluation of the organization and order in the vaccination centers arises as a citizen demand and response to the various episodes of normative transgression traditionally observed in Peruvian society, which were aggravated during the pandemic (31). In other words, a positively evaluated quality public service which place the citizenry at the center of the State’s priorities, could be a tool for public institutions’ legitimacy because it strengthens the institutionality (57), and, consequently, could be the beginning for modeling a socially responsible response in citizens (see (61)).

More specifically, although most aspects of satisfaction with the vaccination service are related to a better balance of emotions, the regression analyses carried out show that the increase in positive emotionality would focus mainly on three central aspects (1) the cleanliness of the facility, (2) the rapidity of attention and (3) the security of the facilities; consistently with this, the mitigation of negative emotions would be related to (1) the cleanliness of the facility and (2) the agile attention. Interestingly about these results is that people increase their emotional well-being after these conditions described before, which apparently could be perceived as services’ basic levels, but notorious in a country with deficient public health services. They had a positive impact on their emotional well-being, just by including an inclusiveness policy and bringing quality service without discrimination by any social category. This could be the tipping point for a new care standard and model in public services for all citizens. Considering the above, it is not surprising that the highest levels of satisfaction are associated with one of the establishments accessed by the poorest citizens in Lima.

Despite the country’s poor performance in dealing with the sanitary emergency as a consequence of the structural problems mentioned above, it is important to remark that the incorporation of LEGADO in the government’s strategy to reverse the negative effects of the pandemic was a good decision. LEGADO, from an approach of public value and institutional strengthening, had the capacity to implement complex logistical processes in the centers at its disposal, ensuring the provision of quality services to the general public, with justice and equity. This experience highlights the necessity of introducing social innovation and modernization standards into public administration as part of the path for developing a stronger rapprochement between the State and the people, in order to build trust between them. Furthermore, based on the findings, it is reasonable to believe that satisfaction with the vaccination service and its effect on emotional responses would be an incentive for those who received the service to return for booster doses and promote the vaccination campaign (40, 41). One of the initially major concerns for the government, as well stated.

Finally, it is important to mention that, while this study illustrates LEGADO’s contribution to the vaccination process in Lima, it is not a public health institution. However, as a state institution with responsibilities including the administration of movable and immovable goods as well as the legacy of the Pan American and Parapan American Games Lima 2019, its participation in the vaccination process with infrastructure, human resources, and a management model has demonstrated that the public administration could provide services of quality to the population. In this sense, it is important to systematize the experience derived from LEGADO’s actions in order to build a resilient emergency response system for disaster situations and complex events in the future. Moreover, to install and reinforce a States’ service modeling from citizens’ perspective, needs and demands with inclusiveness, to lead towards the strengthening of institutionality in Perú.

## Data availability statement

The raw data supporting the conclusions of this article will be made available by the authors, under request.

## Ethics statement

Ethical review and approval was not required for the study on human participants in accordance with the local legislation and institutional requirements. Written informed consent to participate in this study was provided by the participants’ legal guardian/next of kin.

## Author contributions

AE and AC-P contributed to the conception and design of the study. AC-P, MT, JL, and NC supervised the data gathering process in LEGADO’s locations. AE, JM, AC-P, and MT organized the database and wrote the first draft of the manuscript. AE, JM, and AC-P performed the statistical analysis. All authors contributed to manuscript revision, read, and approved the submitted version.

## Conflict of interest

The authors declare that the research was conducted in the absence of any commercial or financial relationships that could be construed as a potential conflict of interest.

## Publisher’s note

All claims expressed in this article are solely those of the authors and do not necessarily represent those of their affiliated organizations, or those of the publisher, the editors and the reviewers. Any product that may be evaluated in this article, or claim that may be made by its manufacturer, is not guaranteed or endorsed by the publisher.
